# MRI findings in a dementia center from Recife, Brazil: applicability of the Fazekas’ score

**DOI:** 10.1002/alz.093078

**Published:** 2025-01-09

**Authors:** Francisco Sabino de Oliveira Neto, Lucas Henrique Calixto, João Pedro Wanderley Soares, Pedro Jatobá Arteiro, Alice Batista Gondim, Luziany Carvalho Araújo, Breno José Alencar Pires Barbosa

**Affiliations:** ^1^ Universidade Federal de Pernambuco, Recife Brazil

## Abstract

**Background:**

Data about dementia in Brazil are scarce and subnotified. Until 2018, 1,3 million people were estimated to have been affected. neuroimaging has a crucial importance to provide detailed information about cerebral structures and characteristics patterns of different types of dementia even as the progression of these injuries. One of the most used imaging scales since 1987 is the Fazekas’ scale, which graduate the lesions according to their distribution in cerebral parenchyma. we aimed to analyze the encephalon’s MRI from patients that are in outpatient follow‐up and caracterize main radiology white matter findings.

**Method:**

It’s a transversal, retrospective and descriptive study about long‐sufferings which were in outpatient between 2017 and 2022 in a North East reference service. Were selected those which the MRI has been done in this service and evaluated by a neuroradiologist using the HORUS™ image analysis software, determining radiologic patterns using Fazekas’ scale

**Result:**

Among ninety‐nine medical records evaluated, there were 26 CT Scans, 15 PET‐CT and 76 MRIs. From this exams, only 35 patients had MRI scans at this service. Two patients were excluded from the study because of the incomplete sequences, with no possibility of evaluation. About the 33 remaining patients, 3 of them had incomplete image sequences, but with an evaluable flair. The mean age was 64.9 years old (+/‐ 12.9), which 57% were female. About the associated comorbities, there were 18 cases of Hypertension (ASH), 4 of diabetes mellitus (DM), 9 of alcoholism and 10 of smoking. The Fazekas Score in these patients were: 0 in 2 patients, 1 in 14 patients, 2 in 9 patients and 3 in 8 patients

**Conclusion:**

Although it’s an old instrument, the Fazekas’ scale is still an actual and with considerable applicability way to graduate lesions in patients with dementia and risky factors using of a simplified way.

